# Ectopic Cervical Deciduosis: A Rare Cause of Antepartum Hemorrhage in Mid Trimester

**DOI:** 10.5152/eurasianjmed.2021.20163

**Published:** 2021-06

**Authors:** Mishu Mangla, Ruchira Nautiyal, Nadia Shirazi, Banishree Pati

**Affiliations:** 1 Department of Obstetrics & Gynaecology, All India Institute of Medical Sciences, Bibinagar, Hyderabad, India; 2 Department of Obstetrics & Gynaecology, Swami Rama Himalayan University, Dehradun, Uttarakhand, India; 3 Department of Pathology, Swami Rama Himalayan University, Dehradun, Uttarakhand, India

**Keywords:** Placentation, antepartum haemorrhage, polyps

## Abstract

Decidual change is a key process required by the uterus to make itself ready for implantation. Presence of ectopic decidual tissue outside the uterine cavity is known as deciduosis. The clinical presentation can vary from being totally asymptomatic and subtle to presenting in the form of life-threatening emergencies like hemoperitoneum, recurrent pneumothorax, or even bowel perforation. Here, we present a case of ectopic deciduosis of cervix presenting in the form of severe life-threatening antepartum hemorrhage in second trimester of pregnancy.

## Introduction

Decidual change is a key process required by the uterus to make itself ready for implantation. Presence of ectopic decidual tissue outside the uterine cavity is known as deciduosis. Progesterone is the predominant hormone responsible for the growth of such tissues; therefore, apart from pregnant women, these tissues are also found in women on exogenous progesterone therapy. Although ovary and cervix are the most common sites, ectopic decidualization has also been reported to occur in other sites including peritoneal cavity,^1^ appendix and bowel,^2^–^4^ or even in the uterine cavity in the form of atumor like mass.^5^ The clinical presentation can vary. Here, we report a case of ectopic cervicaldeciduosis presenting in the form of severe life-threatening antepartum hemorrhage in second trimester of pregnancy.

## Case Report

**Figure 1. a–c:**
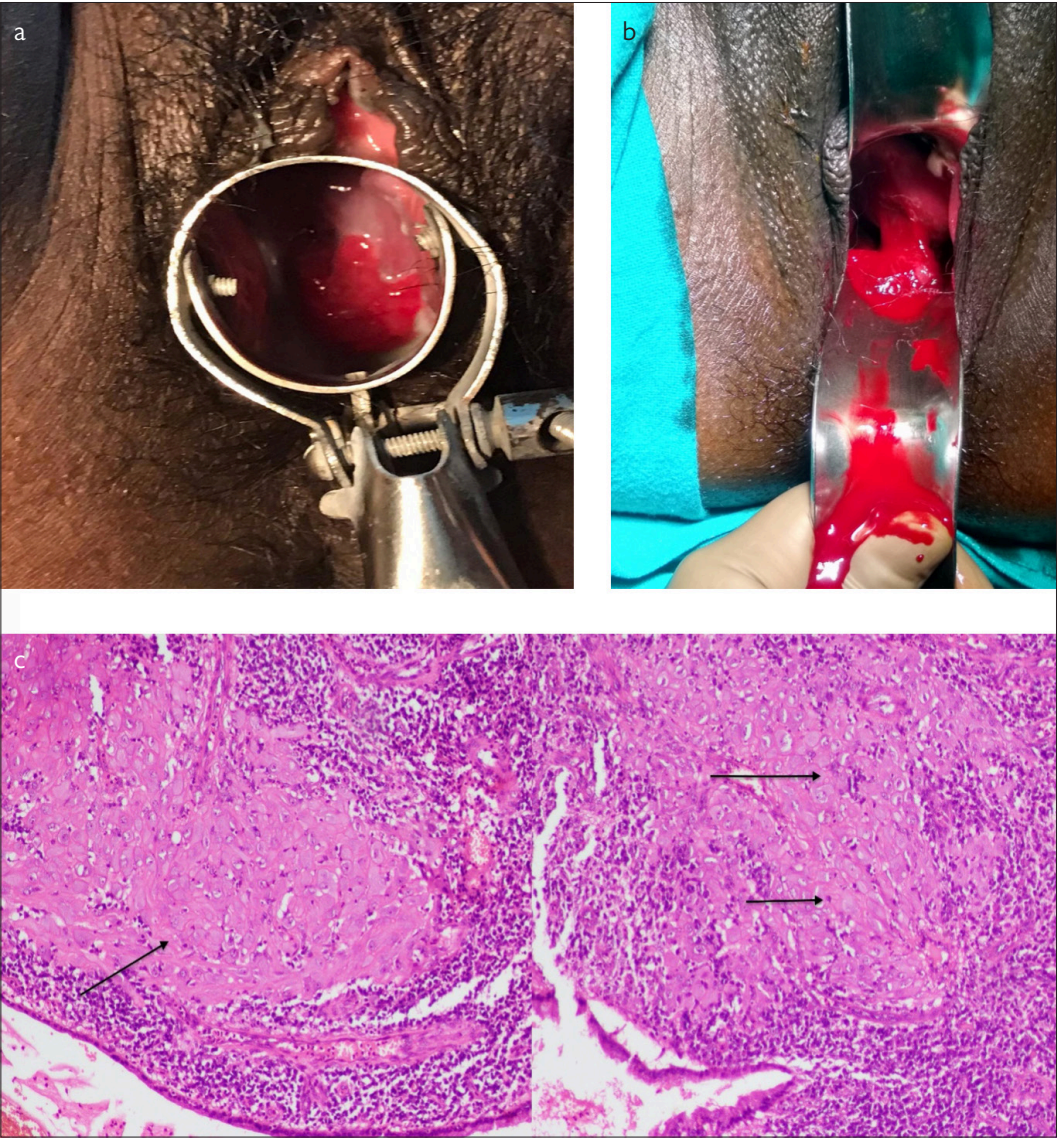
(a) Cervical polyp as seen at 20 weeks, causing spotting, managed conservatively, (b) Cervical Decidualised polyp at 24 weeks presenting with ante partum haemorrhage, (c) H&E:10x:10X: Sheets of decidualized tissue (arrow) surrounded by dense chronic inflammatory infiltrate in cervix (left), H&E:20X10X: Sheets of decidualized tissue (arrows) in cervix (Right) 249x269mm (144 x 144 DPI)

A 28-year-old primigravida, at 24th week of gestation period, presented to obstetrics emergency department with complaint of excessive bleeding per vaginum from last 4 hours. Bleeding was insidious in onset, but later became continuous with passage of clots. She had soaked 4 pads in last 4 hours. Bleeding was bright red in color and was not associated with any abdominal pain or tightening of uterus. There was no history of high blood pressure her antenatal period. There was no history of abdominal trauma or pelvic instrumentation and no history of exogenous progesterone therapy. She had experienced 2 episodes of spotting at 16 weeks and 20 weeks, respectively, which were managed conservatively. A per speculum examination done at 20 weeks showed a cervical polyp arising at the 4 o’clock position (Figure 1a). It was managed conservatively, as it was not bleeding actively.

On examination, she was found to be conscious, oriented to time, place, and person but extremely pale. Her pulse was 120 beats per minute (bpm), blood pressure was 80/50 mm Hg, and respiratory rate was 20/min. On abdominal examination, uterus corresponded to 24 weeks of pregnancy; it was relaxed, nontender and nontense. Fetal heart sound was 140 bpm regular. On local examination, active bleeding which was bright red in color and painless was observed. A low-lying placenta was already ruled out by her Level II scan. A repeat ultrasound confirmed the previous findings of fundal attachment of placenta and no signs of retroplacental clot or hemorrhage. A gentle per speculum examination was performed. After removing approximately 250 ml of blood clots from the vagina, a bright red polyp measuring 2x2 cm was observed at the 4 o’clock position of cervix (Figure 1b). As it was bleeding actively, polypectomy was performed under general anesthesia after stabilizing the patient. Hemostatic sutures were applied at the base of the polyp. She required 2 units of blood transfusion. She recovered well in the postoperative period. She delivered a live fetus by full term vaginal delivery at 39 weeks of gestation.

The histopathology report of polyp showed sheets of decidualized tissue surrounded by dense chronic inflammatory infiltrate in cervix suggestive of cervical deciduosis (Figure 1c).

## Discussion

Decidualization is an integral part of implantation and placental development. If it occurs at any site, other than the uterus, it is said to be ectopic. Although cervix and ovaries are the most common sites, it has been reported to occur in vagina, peritoneum, and even in the uterine cavity in the form of tumor-like mass.^5^,^6^ Rare sites include kidney, lungs, and skin.^7^–^9^

Ectopic deciduais usually a microscopic finding detected on the histopathology reports of biopsy taken during caesarean section, postpartum tubal ligations, or in tubal ectopic.^6^ Although, the condition is very common, it is usually asymptomatic.^10^ Buttner et al.^11^ reported that in their study, 100% of omental biopsy specimens were positive for decidual cells. Some form of macroscopic ectopic decidual tissue has been reported to be present in approximately 10% of patients who underwent caesarean section. Oftentimes, peritoneal tubercles are observed at the time of caesarean section, especially on the posterior surface of uterus and ovaries. These, if biopsied, might be subtle manifestations of deciduosis themselves. Rarely, however, they have been reported to present in the form of life-threatening complications such as hemoperitoneum, obstructed labor, pulmonary involvement, or even perforation of a hollow viscera. Our case was unique in this regard, as the decidual reaction of cervical polyp presented through a life-threatening antepartum hemorrhage.

Various theories have been proposed regarding the origin of deciduosis. It is presently believed that decidual change in cervix is due to abnormal reaction of normal stromal cells to hormonal stimulation of pregnancy, especially progesterone.^12^ Ectopic decidua at other sites such as peritoneal cavity and omentum are due to metaplasia of superficial coelomic stroma in response to progesterone.^13^

Cervical deciduosis usually presents in the form of small sessile polypsor small elevated, single or multiple, highly vascular nodules.^14^ Lesions as big as 8 cm have been reported in literature.^15^ However, in this case, it presented in the form of a highly vascular pedunculated polyp(Figure 1a, 1b). A high degree of suspicion is a must for the diagnosis of such cases as these polyps may even look like neoplastic masses. This highlights the importance of a good perspeculum examination to rule out local causes of antepartum hemorrhage.

Although deciduosis is a benign cervical change in pregnancy and usually undergoes resolution spontaneously within 4–6 weeks postpartum, it should be kept in the differential diagnosis of local causes of antepartum hemorrhage.

## References

[1] Sabatelle R, Winger E (1973). Postpartum intraabdominal hemorrhage caused by ectopic deciduosis. Obstet Gynecol.

[2] Malpica A, Deavers MT, Shahab I (2002). Gross deciduosis peritonei obstructing labor: a case report and review of the literature. Int J Gynecol Pathol.

[3] Bloom SL, Uppot R, Roberts DJ (2010). Case records of the Massachusetts General Hospital. Case 32-2010. A pregnant woman with abdominal pain and fluid in the peritoneal cavity. N Engl J Med.

[4] Gradauskas A, Činčikas J, Daunoravičius R (2012). Ectopic decidua presenting with a sigmoid bowel perforation: a case report. J Clin Case Rep.

[5] Dasani M, Lee HJ, Rijhsinghani A (2019). Deciduoma, a large intrauterine mass of deciduosis. AJP Rep.

[6] Shukla S, Pujani M, Singh SK (2008). Ectopic decidual reaction mimicking peritoneal tubercles: a report of three cases. Indian J Pathol Microbiol.

[7] Bettinger HF (1947). Ectopic decidua in the renal pelvis. J Pathol Bacteriol.

[8] Flieder DB, Moran CA, Travis WD, Koss MN, Mark EJ (1998). Pleuro-pulmonary endometriosis and pulmonary ectopic deciduosis: a clinicopathologic and immunohistochemical study of 10 cases with emphasis on diagnostic pitfalls. Hum Pathol.

[9] Fair KP, Patterson JW, Murphy RJ, Rudd RJ (2000). Cutaneous deciduosis. J Am Acad Dermatol.

[10] Markou GA, Goubin-Versini I, Carbunaru OM, Karatzios C, Muray JM, Fysekidis M (2016). Macroscopic deciduosis in pregnancy is finally a common entity. Eur J Obstet Gynecol Reprod Biol.

[11] Burnett RA, Millan D (1986). Decidual change in pelvic lymph nodes: a source of possible diagnostic error. Histopathology.

[12] Chapman GW, Savage EW, Salem FA (1979). Cervical deciduosis and intraepithelial neoplasia. J Natl Med Assoc.

[13] Zaytsev P, Taxy JB (1987). Pregnancy-associated ectopic decidua. Am J Surg Pathol.

[14] Van Diepen DA, Hellebrekers B, van Haaften AM, Natté R (2015). Cervical deciduosis imitating dysplasia. BMJ Case Rep.

[15] Gornall AS, Naftalin NJ, Brown LJ, Konje JC (2000). Massive necrosis of cervical ectopic decidua presenting in labour. BJOG.

